# Genetics of infertility and “assisted fertilization” in the Bible: The case of Abraham and his family

**DOI:** 10.1111/andr.70103

**Published:** 2025-08-01

**Authors:** Manuela Simoni, Frank Tüttelmann, Livio Casarini

**Affiliations:** ^1^ Department of Biomedical Metabolic and Neural Sciences Baggiovara Hospital University of Modena and Reggio Emilia Modena Italy; ^2^ Unit of Endocrinology Department of Medical Specialties Azienda Ospedaliero‐Universitaria of Modena Modena Italy; ^3^ Institute of Reproductive Genetics Centre of Medical Genetics University of Münster Münster Germany

**Keywords:** Abraham, Bible, evolutionary, medicine, PCOS

## Abstract

Couple infertility is a very ancient medical condition. One of the first descriptions of familial infertility/subfertility is contained in the first book of the Bible, Genesis, written in the 10th century BC and reporting tales from the oral tradition even occurred about 800 years earlier. Genesis reports the peculiar case of Abraham and his descendants, Isaac and Jacob, the three patriarchs of Israel. The (favorite) wife of the three of them suffered from primary infertility and, according to Genesis, could reach pregnancy only in late age and for divine intervention. In this paper, we reconstruct the pedigree of Abraham's family on the basis of the biblical narration, which is rich enough of medical details to allow to reconstruct a plausible medical/genetic reason for the cases of infertility reported therein.

## 1 INTRODUCTION

The history of the patriarchs of Israel represents the founding basis of monotheistic religions. It is largely legendary and derives from very ancient oral traditions typical, in various forms and literary genres, of the whole “Fertile Crescent” of the Middle East. It is narrated in the book of Genesis, the first book of the Bible and of the Pentateuch, the Jewish Torah. Genesis tells the story of Abraham, the founding father of the nation of Israel as well as of the Arab people, and the patriarch recognized by Jews, Muslims, and Christians. The peculiarity of this story is that the (favorite) wives of Abraham and his descendants were invariably sterile, a contradiction in terms for the lineage that founded different peoples. Abraham's family has demonstrated the importance of fertility/infertility since very ancient times and is an example of a consistent biblical theme where infertility is often met with divine intervention leading to miraculous conceptions. In the Bible, it is the faith in God's promises that allows conception, which is viewed as a gift from God rather than a merely biological process.

Apart from Genesis, the Bible reports in various books stories of sterile women, who managed to have children through divine intervention. These children then turned out to be leading figures in the history of Israel and Salvation. Beyond the religious and messianic interpretations, in this article, we summarize the main examples of sterility reported by the Bible and attempt a secular and scientific reflection on the peculiar form of familial infertility of Abraham's family.

## 2 STORIES OF INFERTILITY IN THE BIBLE

Some introductory remarks are necessary before going into details. The marriage and reproductive customs of ancient biblical times were very different from ours. By comparison, our times are rather sanctimonious. Polygamy was the norm, as still is in some Semitic populations. Children born to the husband with another woman, given to him for this purpose by the barren wife, were considered her own, as a kind of surrogate womb. This was because sterility was always a female problem, never the husband's fault, and often considered the result of some sin committed by the woman or her ancestors. In addition, marriages within the same family were (and still are in some Semitic populations) sought after and preferred, in order to maintain the integrity of the assets and wealth. Finally, the guest was sacred to the point of offering him the women of the own house for the night.

Synthetically, here we list the most prominent examples of sterile women in the Bible (Summary produced with the aid of the generative AI tools, Perplexity, ChatGPT4.0, and Claude 3.5 Sonnet, prompted on 16.1.25 with the query: “Retrieve and discuss (with accurate bibliographic references) all examples or instances (even mentions) of infertile women (or couples) who at some point conceived a child in the Bible.”).

### 2.1 Sarah (mother of Isaac)

Sarah was the wife of Abraham and remained childless until the age of 90. Her story is told in Genesis, particularly chapters 16–21. The text explicitly states “Now Sarah was barren; she had no child” (Genesis 11:30). God promised Abraham that Sarah would bear a son despite her advanced age and previous infertility. Initially, Sarah laughed at this promise, considering it impossible (Genesis 18:12). However, she eventually conceived and bore Isaac, whose name means “laughter,” a reference to both Sarah's initial disbelief and later joy.

### 2.2 Rebekah (mother of Esau and Jacob)

Isaac's wife Rebekah was also initially unable to conceive. Genesis 25:21 tells us “Isaac prayed to the Lord on behalf of his wife, because she was childless.” After 20 years of marriage, following Isaac's prayers, she conceived twins, Esau and Jacob.

### 2.3 Rachel (mother of Joseph and Benjamin)

In Genesis 29‐30, we find the story of Rachel, Jacob's beloved wife. The text specifically notes “When the Lord saw that Leah was not loved, he enabled her to conceive, but Rachel remained childless” (Genesis 29:31). Rachel's infertility caused her great distress, particularly as she watched her sister Leah bear multiple children. After years of infertility, “God remembered Rachel; he listened to her and enabled her to conceive” (Genesis 30:22). She gave birth to Joseph and later to Benjamin.

### 2.4 Manoah's wife (mother of Samson)

In Judges 13:1‐24, we learn about Samson: “The Israelites offended the Lord, who therefore delivered them into the power of the Philistines for forty years. There was a certain man from Zorah, of the clan of the Danites, whose name was Manoah. His wife was barren and had borne no children. An angel of the Lord appeared to the woman and said to her, though you are barren and have had no children, yet you will conceive and bear a son. Now, then, be careful to take no wine or strong drink and to eat nothing unclean. As for the son you will conceive and bear, no razor shall touch his head, for this boy is to be consecrated to God from the womb. It is he who will begin the deliverance of Israel from the power of the Philistines.”

Samson was a judge of Israel and with his death freed Israel from the domination of the Philistines.

### 2.5 Hannah (mother of Samuel)

The story of Hannah is recorded in 1 Samuel 1‐2. She was one of two wives of Elkanah, and the text notes that “the Lord had closed Hannah's womb” (1 Samuel 1:5). Her fervent prayer at the temple in Shiloh, and her vow to dedicate any son to God's service, precedes God's intervention. She eventually conceived and bore Samuel, who became a significant prophet in Israel.

### 2.6 Shunammite woman

In 2 Kings 4:8‐17, we find the story of a wealthy Shunammite woman who showed hospitality to the prophet Elisha. Although not explicitly described as infertile, she had no child and her husband was old. Elisha prophesies that she will have a son, and despite her skepticism (“No, my lord! Don't mislead your servant, O man of God!”), she conceived and bore a son. Unfortunately, the son died as a young boy, but Elisha was able to revive him with a maneuver similar to cardiac massage and mouth‐to‐mouth resuscitation (2 Kings 4:33‐37).

### 2.7 Elizabeth (mother of John the Baptist)

In the New Testament, we encounter Elizabeth, wife of the priest Zechariah. Luke 1:7 explicitly states that “they were childless because Elizabeth was not able to conceive, and they were both very old.” The angel Gabriel announced that she would bear a son. Through divine intervention, she conceived and bore John the Baptist, who became the forerunner of Jesus. Of note, the angel Gabriel announced, at the same time, the miraculous, virginal conception of Jesus to Mary, Elizabeth's cousin. However, Mary was probably fertile because the gospels talk about Jesus’ brothers and sisters; for example, Mark 6:3 says that the people who were listening to Jesus in the synagogue were saying “Is he not the carpenter, the son of Mary, and the brother of James and Joses and Judas and Simon? And are not his sisters here with us?”

Sarah, Rebekah, and Rachel belong to the Abraham's family, and are characterized by a “familial” form of female infertility. Setting apart the divine intervention, which could be considered an ancient and magic form of “assisted reproduction,” and regardless of the obvious legendary nature of the story, we make here an archeo‐pathophysiological exercise and consider whether this condition might have had some medical reason and what could be the mechanism of eventual fertility after many years of barrenness in the women of Abraham's family.

## 3 REPRODUCTIVE HISTORY OF ABRAHAM'S FAMILY

### 3.1 Abraham

The saga of Abraham's family took place in the Bronze Age (about 1800 years BC), but it was only during the reign of Solomon (971‒931 BC) that the history of the patriarchal traditions, until then transmitted orally, began to be written. The Torah takes on its current literary form only in the fifth century BC. Therefore, what we read in Genesis certainly does not constitute a medically accurate account of what had perhaps happened over a 1000 years earlier. The text does, however, offer some important hints for attempting a secular, medical interpretation of the events.

**FIGURE 1:**
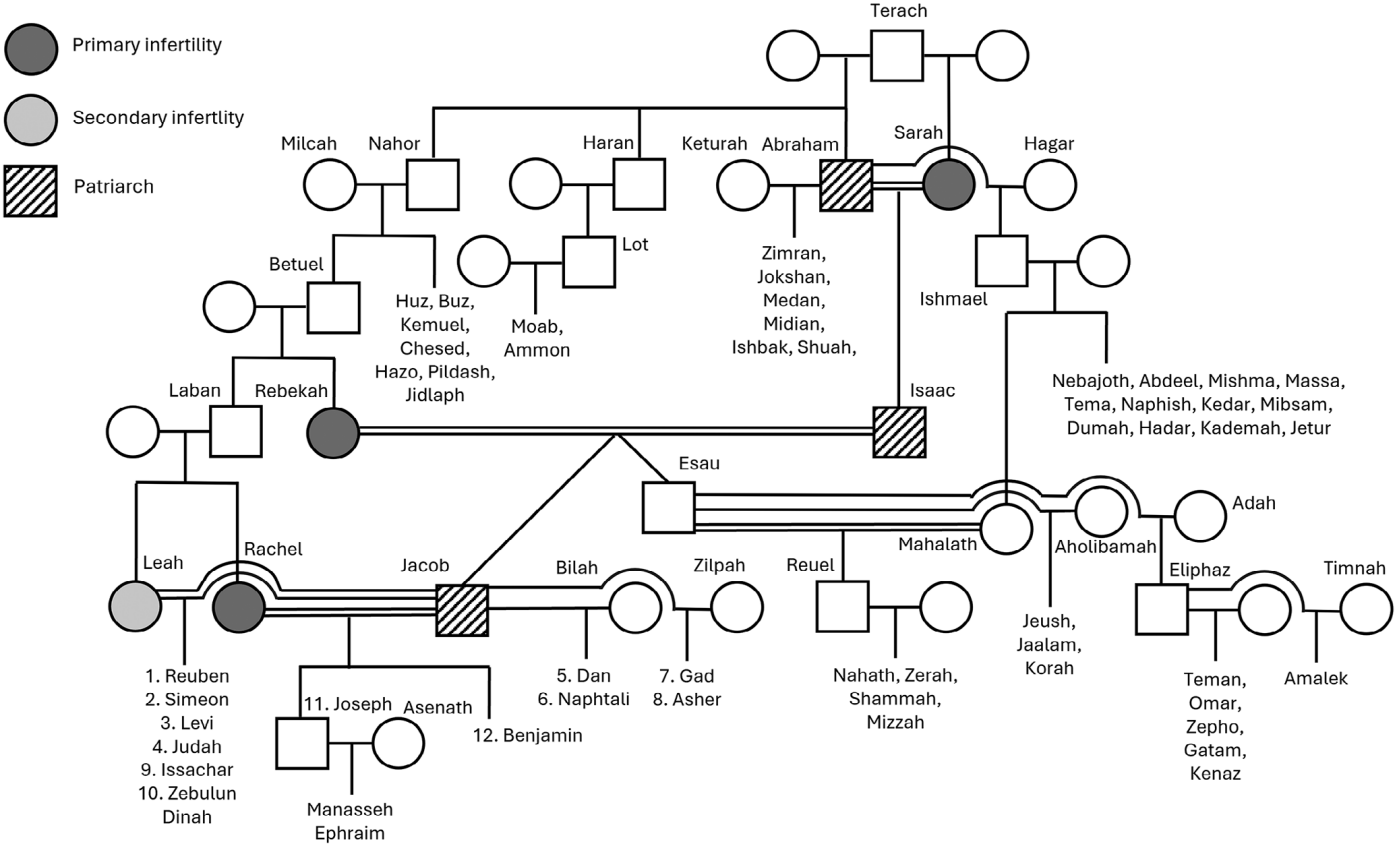
Terach's family tree. Males are indicated by squares, whereas females by rounds. Double lines show consanguinity between partners, and numbers indicate the founder of tribes of Israel.

Abraham was one of the three sons of Terach (Figure 1) and took Sarah as his wife. Sarah was daughter of the same father, Terach, with another woman, so that the two were stepsiblings. Genesis 11:30 says specifically that Sarah was sterile. At this point, the family emigrates from Mesopotamia (Ur of the Chaldeans) toward the Land of Canaan, the today's Palestine/Israel region, by order of God, who promises Abraham the territories and to become the founder of a great nation. A famine forces Abraham to migrate further toward Egypt, where he says around that Sarah was his sister: he did so because, in spite of being already 65 years old, Sarah was a very pretty woman and he was afraid to be killed had he said that she was his wife. After all, it was not a lie that Sarah was his sister. The Pharaoh was captivated by Sarah's beauty, took her as his wife, and rewarded Abraham with many possessions, including cattle and slaves. When the Pharaoh discovered that Sarah was Abraham's wife, he chased him away from Egypt with all his goods because it was a sacrilege to take the wife of another alive man. In this way, Abraham became very wealthy and went back to Palestine, settling in Hebron. From this episode, we take the hint that *Sarah and not Abraham was the infertile* member of the couple; otherwise, assuming that Sarah was not menopausal yet (the detail that she was *still very attractive* could be a sign of estrogenization), she could have generated children with the Pharaoh, as it is described, that menopause can occur at very old age, even after 65.^1^

At this point, God promises again Abraham that he will give him descendants as numerous as the stars in the sky but Sarah continued to be sterile. It is Sarah herself who proposes a solution: (Genesis 16) “The Lord has kept me from bearing children. Have intercourse, then, with my maid; perhaps *I shall have sons through her*.” Abraham heeded Sarah's request and took Hagar as his concubine: Hagar bore him a son and Abraham, who was 96, called him Ishmael. From this episode, we learn that *Abraham was fertile* and that, according to the use of the time, a “surrogate womb” approach was taken to overcome couple infertility. The case of Abraham and Sarah/Hagar is the first description of surrogacy in history, although quite different from what we intend today by this term. In the time of Genesis, the woman was believed to generate offspring from semen only, with no knowledge about the existence of male and female gametes. Therefore, it was ethically unimportant which womb was used to produce a baby and no direct information about Sarah's ovarian function can be found in the text.

Three years later, Abraham was 99 and the Lord promises again (Genesis 17) “As for your wife […] Sarah […] I will bless her, and I will give you a son by her. Him also will I bless; he shall give rise to nations, and rulers of peoples shall issue from him.” At this point, some perplexity about fertility in such old age arises and Abraham thought “Can a child be born to a man who is a hundred years old? Or can Sarah give birth at ninety?” Never mind: at this point (Genesis 18), three men appear, are hosted with all honors by the couple and make an announcement. “One of them said, ”I will surely return to you about this time next year, and Sarah will then have a son. Genesis continues: “Now Abraham and Sarah were old, advanced in years, and *Sarah had stopped having her womanly periods*.” From this, we learn that the cause of Sarah sterility in younger age *was not because of amenorrhea*. In chapter 21, Genesis informs us that “Sarah became pregnant and bore Abraham a son in his old age … Abraham gave the name Isaac to this son of his whom Sarah bore him.”

After the birth of Isaac, Sarah, now physical mother, became jealous of Hagar and Ishmael, the firstborn, and Abraham was forced to send them away to the Desert of Paran (today's Negev). Later on, Ishmael will generate 12 sons from an Egyptian wife and will become the founder of the Arab outrages (Figure 1).

At the age of 127, Sarah passed away. After Sarah's death, Abraham took another wife, Keturah, from whom, he generated six more children, another indication that he was fertile. At his death, when he was 175 years old, all his possessions were left to Isaac.

### 3.2 Isaac

Abraham had two brothers (Figure 1). One of them, Nahor was the father of Bethuel, who, in turn, was the father of Laban and Rebekah. Genesis’ chapter 24 recounts in detail how Abraham sent his servant to his country of origin to find a wife for Isaac. The servant reached Haran, where he found Rebekah, the daughter of Bethuel, who was Abaham's nephew. The servant negotiated the terms of marriage with Bethuel and Rebekah's brother Laban and paid a rich dowry to them. When all negotiations were concluded, Rebekah was consulted and she accepted according to the rules of times when the woman's opinion was formally asked but basically irrelevant. In this way, Isaac married a young virgin from the same clan. In fact, in old biblical times, people were married in early youth, and marriages were usually contracted within the narrow circle of the clan and the family. It was undesirable to marry a woman from a foreign clan, lest she introduce foreign beliefs and practices. This resulted in consanguinity, which might be related to higher fertility even in contemporary primitive tribes.^2^

So, at the age of 40, Isaac married Rebekah when she was a very young girl: the two were second cousins (Figure 1). Rebekah was infertile. Isaac (Genesis 25) “entreated the Lord on behalf of his wife, as she was sterile. The Lord heard his entreaty, and Rebekah became pregnant.” At that point, Isaac was 60; therefore, the couple *experienced 20 years of primary infertility before becoming pregnant*. Genesis (25) informs us that at delivery “The first to emerge was reddish, and his whole body was like a hairy mantle; so, they named him Esau. His brother came out next, gripping Esau's heel; so, they named him Jacob.” From this tale, we pick the information that Isaac and his wife suffered from primary couple infertility, which lasted 20 years, after which *a dizygotic twin pregnancy occurred*. Genesis clearly states that it was Rebekah who was sterile, but *Isaac was son of consanguineous parents* (Figure 1) so we cannot exclude some genetic factor affecting spermatogenesis. The non‐identical twin pregnancy means *double ovulation when Rebekah is at least beyond 30–35 years of age*. Genesis does not report about other wives or concubines of Isaac, who, apparently, generated only these two sons with Rebekah. This being uncommon for the times, we could speculate some kind of *subfertility* in Isaac.

Esau and Jacob were always antagonists: Esau was the firstborn, a man of action, a hunter and the favorite of his father Isaac, while Jacob was a quiet, smart man and the favorite of his mother Rebekah. In his late age, *Isaac went blind* and could not distinguish between the sons by sight. Taking advantage of this, Rebekah helped Jacob to deceive his father with a stratagem, tearing away from him the blessing reserved for the firstborn. Esau lost the birthright and persecuted Jacob who had become his superior. To save Jacob from being murdered, Rebekah sent him away from home to her brother Laban.

Esau then had three wives, one of them, Mahalath, was the daughter of Ishmael and thereby his cousin. He generated several descendants and is considered the founder of the Edomites. Mahalath is not told to be infertile but she had only one child, which is uncommon for the times and the family.

### 3.3 Jacob

On his way to Haran, the Lord appeared to Jacob in a dream, identified himself as the “God of Abraham and Isaac” and promised: “the land on which you are lying I will give to you and your descendants. These shall be as plentiful as the dust of the earth, and through them you shall spread out east and west, north and south.” Once in Haran, Jacob met a young shepherdess by a well, the younger daughter of his uncle Laban, Rachel. Her older sister was Leah. Genesis 29 reports that “Leah had lovely eyes, but Rachel was well formed and beautiful.” So, Jacob fell in love with Rachel and told Laban “I will serve you 7 years for your younger daughter Rachel.” Laban agreed but, at the end of the 7 years of service and after the wedding banquet, he deceived Jacob and “at nightfall he took his daughter Leah and brought her to Jacob, and Jacob consummated the marriage with her.” This was because “it is not the custom […] to marry off a younger daughter before an older one.” However, “finish the bridal week for this one, and then I will give you the other too, in return for another 7 years of service with me”: two wives, two dowries.

Therefore, Jacob married his two first cousins. Rachel was his favorite, but she was sterile. On the contrary, Leah, in the attempt to capture the preference of the husband, generated four sons. Rachel became envious of her sister's prolificity and gave Jacob her maidservant Bilhah as a consort with the task to impregnate her (Genesis 30) and “let her give birth on my knees, so that I too may have offspring, at least through her.” Bilhah bore two sons. At this point, Leah, who meanwhile “stopped bearing” (Genesis 29:35) and was unable to conceive further, gave also her maid Zilpah to Jacob as a concubine, so that more children could be born under her name. Zilpah bore two more sons to Jacob.

After these events, Leah, being unhappy about her inability to procreate further, thought about using some medicinal remedy and, “during the wheat harvest,” sent her first son Reuben to the fields to find some mandrakes. Reuben indeed brought home the mandrakes to his mother but Rachel insisted to take possession herself of such plants with fertility‐enhancing properties. Since Leah now suffered also of infertility, Rachel made a deal with the sister and traded the mandrake for her consent for Leah to sleep with Jacob again. Leah accepted and, irony of fate, on that very evening conceived another son from Jacob and, in the following years, again another son and a daughter. Obviously, mandrake did not work with Rachel, and Leah was fertile again without medicines.

Eventually (Genesis 30, 22), “God remembered Rachel; he heard her prayer and made her fruitful.” So, she conceived and bore a son, Joseph. After this event, Jacob left Laban's land, not without having stolen from him many riches and herds with stratagems and tricks, and returned to his homeland. He then departed again with the family and on the way from Bethel to Ephrath (today's Bethlehem), Rachel delivered her second and last son, Benjamin, and died in childbirth. This was the 12th son of Jacob. Assuming that all Jacob's children had born in sequence with roughly yearly frequency, and that Rachel was a teenager when she married, she must have been around 35 when she became fertile. This is confirmed by Jacob's statement that he served 20 years in Laban's house before leaving (Genesis 31, 38 and 31, 41).

The 12 sons of Jacob, who were renamed Israel by God, gave rise to the 12 tribes of Israel.

From this story, we learn that *both primary* (Rachel) *and secondary* (Leah) *infertility afflicted Jacob's wives*, while he himself was clearly very *fertile in spite of being son of consanguineous parents*. As in the case of Sarah and Abraham, the “surrogate womb” solution demonstrates the fertility of men in this family, with the possible exception of Isaac's *subfertility*. On the other hand, Leah is a case of *secondary infertility, which, in addition, is reversible*. Rachel became fertile at around 35 years of age and we know that, at the time when they left Laban's house, *she had menstruations*, as she warned the father (Genesis 31, 35) “Let not my lord feel offended that I cannot rise in your presence; a woman's period is upon me.” Furthermore, we learn about the *aphrodisiac and fertility‐enhancing properties of mandrake* for the first time in the world literature.^3^

## 4 INTERLUDE: MANDRAKE AND FERTILITY

**FIGURE 2:**
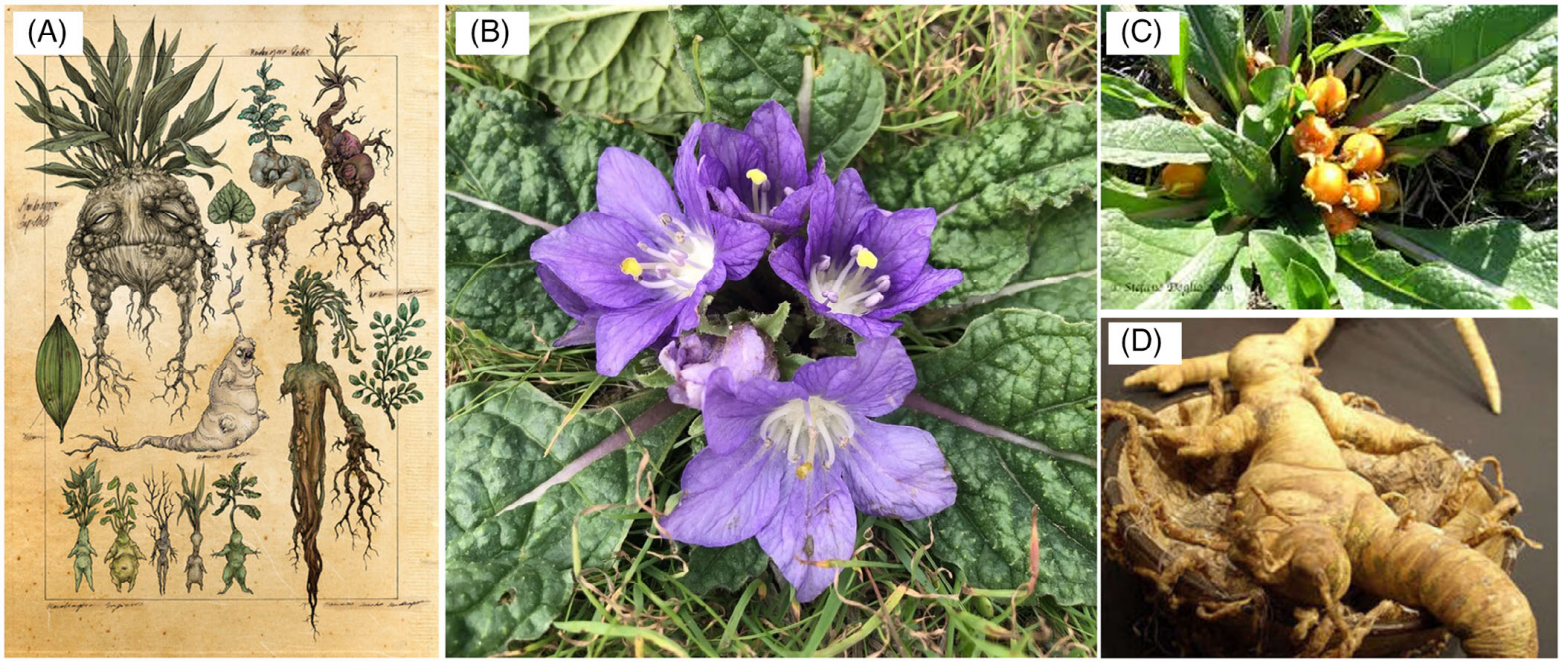
*Mandragora officinarum*: (A) popular representations of the plant's parts, with its anthropomorphic features, (B) flowers, (C) berries, and (D) roots of the plant.

The Hebrew word used to indicate the “love apple” found by Reuben in Genesis 30, 14 is *Dūdā īm*, which means *the pleasure of love* or *carnal love*,^4^ and the fruit is thought to correspond to and was translated into *mandrake*, being this the most probable plant with aphrodisiac and fertility‐enhancing properties that can be found and produce berries in late spring (“at the time of wheat harvest”) in that Mediterranean area. Mandrake (*Mandragora officinarum*, sp. *Solanaceae*, Figure 2) is probably the best‐known medicinal plant with such properties of the ancient times and was used for many medical purposes for centuries. Its use is well documented in the medical history in the scripts of *Hippocrates*, *Aretaeus*, *Celsus*, *Apuleius Platonicus*, and many others.^4^ In the humanistic literature, *mandragora* is mentioned in many works, for example, Shakespeare's *Romeo and Juliet* and *Othello*, Machiavelli's *La Mandragola*, and Beckett's *Waiting for Godot* up to the Rowling's *Harry Potter* saga (In “Harry Potter and the Chamber of Secrets,” Professor Sprout is reported to have taught the second‐year students how to repot young Mandrake plants. Later, when Mrs Norris was found petrified by the Basilisk, the Mandrake Potion was mentioned by Albus Dumbledore as a possible remedy, and on that occasion, it was Professor Lockhart who specifically spoke of it, claiming that he “could make it with his eyes closed.” When Harry Potter clashed with Tom Riddle in the Chamber of Secrets, the young wizard stated that the Mandrake Potion would be ready in a short time and that it would bring back to life all those who had been petrified and, unlike what had happened years before, no one would die because of the Basilisk that had been released. The plant is also mentioned in “Harry Potter and the Goblet of Fire” when Harry, Ronald Weasley and Hermione Granger were browsing through books in the library looking for something that could help the Chosen One pass the second task of the Triwizard Tournament [https://www.potterpedia.it/?v = Mandragola#ixzz8xyo44r5i; last visited 21.1.25]).

A very good, recent paper^3^ compiled and reviewed the medicinal use of mandrake across the centuries in various countries and according to the chemistry and pharmacology of the plant, finding information about as many as 88 medical uses. Chemically, the most significant, among many others, alkaloids contained in mandrake's roots are scopolamine and atropine with parasympatholytic properties. These alkaloids were recently found using very sensitive, high‐resolution mass spectrometry in human hair form archeological excavations in a Bronze Age burial and cult cave in the Balearic Islands,^5^ suggesting that, in Abraham's times, humans were indeed using medicinal plants.

In the “reproductive” area, mandrake extracts have been used for abortion, infertility, as aphrodisiac, and for “female problems” in general. In their review, Benitez et al. mentioned that the aphrodisiac properties of the biblical mandrake were probably given by the scent of the fruits, not the roots, and that such fruits are still collected today for the same purpose in Spain, Turkey, Armenia, Iraq, Lebanon, and Morocco. It would be the particular provocative fragrance of the fruit that causes sexual attraction. However, Dioscorides’ *De materia medica* (first century AC) mentioned the roots having aphrodisiac properties and it is reported that orthodox Jews and Arabs still collect mandrake's fruits to overcome bareness (ref. [3] and references therein). The fertility‐promoting properties of mandrake were mentioned by Machiavelli in the comedy *La mandragola*, in which doctor Callimachus states “You must understand this, that there is nothing more certain to impregnate a woman than to give her a potion made of mandrake.” Still today, with little Google search, anyone can land on sites offering (homeopathic) preparations with mandrake extracts.

Given that mandrake contains parasympatholytic alkaloids, the question arises whether there is any pharmacological basis for the folkloristic belief about the aphrodisiac and fertility‐enhancing properties of the plant. In other words, what is the role of the cholinergic/parasympathetic system in reproduction? In spite of the legendary positive effects of mandrake on sexual activity and fertility, experimental evidence in animals suggests inhibitory effects of anticholinergic alkaloids. In male rats and hamsters, scopolamine inhibits sexual behavior.^6^, ^7^, ^8^ The same effect was described in female rats,^6^ where scopolamine inhibits lordosis,^9^ and rhesus monkeys.^10^ Concerning endocrine gonadal function and fertility, anticholinergic drugs were shown to inhibit fertility in male rats^11^ by reducing the transport of spermatozoa,^12^ to increase follicular atresia in the ovary^13^ and reduce progesterone production in granulosa cells.^14^ Therefore, overall, the effects of mandrake fruits/extracts on fertility should be negative, if any.

In human medicine, anticholinergic drugs that block muscarinic receptors were occasionally used in the past for retrograde ejaculation, for example, in diabetic men.^15^, ^16^, ^17^ Currently, they are used mainly in ophthalmology and as inhaled bronchodilators. Side effects include dry mouth, difficulty in vision and photophobia, difficulty urinating, and even mental confusion in the most serious cases. No effects on sexual behavior or fertility were ever reported.

## 5 WHAT WAS THE REASON FOR INFERTILITY/SUBFERTILITY IN ABRAHAM'S FAMILY? A SECULAR, MEDICAL INTERPRETATION

In Genesis, the three ladies, Sarah, Rebekah, and Rachel, were sterile for the greater glory of divine intervention in favor of the foundation of the people of Israel. Pregnancies became possible because of God's action. With the due respect, we could regard this as a kind of “divinely assisted fertilization” but the question is whether, based on the biblical story, we could identify the medical reason for infertility/subfertility and of the eventual pregnancies at least in some of the cases.

Abraham's pedigree (Figure 1) is remarkable for some characteristics: (1) multiple consanguineous marriages, (2) males are always fertile, (3) the wives of the patriarchs are subfertile (incomplete penetrance?), (4) both primary and secondary female infertility/subfertility are present, (5) infertility does not involve amenorrhea, (6) pregnancies eventually happen at an advanced/very advanced age, and (7) a dizygotic twin pregnancy (double ovulation) occurred. In addition, if we hypothesize a genetic origin of female infertility, it seems that its transmission is through the male germline. The founder is Terach, who generated the infertile Sarah and passed the defect via Nahor to Bethuel, who generated the infertile Rebekah and passed the factor to Laban, who generated the infertile/subfertile Leah and Rachel. This “infertility/subfertility factor” does not affect spermatogenesis and it does not self‐extinguish, suggesting that it might confer some evolutionary advantage. Some endocrine mechanism could enter the field.

The easiest interpretation is a polygenic, familial form of polycystic ovary syndrome (PCOS), reducing fertility in females but without male phenotype. Several loci have been demonstrated to be in association with the PCOS phenotype in genome wide association studies (GWAS) and can be consulted in online catalogs (e.g., https://www.ensembl.org/Homo_sapiens/Phenotype/Locations?db=core;g=ENSG00000125347;ph=6023;r=5:132440440‐132508719).

The most interesting candidate PCOS genes confirmed in independent studies include *DENND1A*,^18^, ^19^, ^20^, ^21^ *FSHB*,^18^, ^19^ *FSHR*,^20^, ^22^ and *LHCGR*.^20^, ^22^ These are all genes well known to be involved in ovarian function.^23^, ^24^, ^25^, ^26^, ^27^ A simple hypothesis could be that polymorphisms or mild inactivating mutations in one or more of these genes could be responsible for a PCOS‐like phenotype in the subfertile women of Abraham's family without producing any reproductive trouble in the males. It is known that inactivating mutations of the *FSHR* do not cause necessarily infertility in males.^28^ Furthermore, PCOS risk has been associated with genetic susceptibility to later menopause (Sarah's case),^19^ and dizygotic twinning (Rebekah's case) was also associated with the *FSHB locus*.^29^ PCOS is also compatible with secondary and transient infertility (Leah's case). Other candidate PCOS susceptibility genes confirmed in independent GWAS include members of the epidermal growth factor receptor genes (*ERBB2/HER2*, *ERBB3/HER3*, and *ERBB4/HER4*),^19^ which are involved in steroidogenesis.

The genetic origin of the Mediterranean PCOS phenotype corroborates the historical descriptions of the condition in ancient Egyptian, Greek, and Roman documents,^30^ and defined in the early Italian medical literature by Antonio Vallisneri in 1721.^31^, ^32^, ^33^ In fact, a previous study demonstrated the existence of a genetic pool, originating from the Ashkenazi and southeast European populations, sharing a certain grade of similarities.^34^ These data are consistent with relatively high PCOS prevalence^35^ and similar phenotype among people from Southern Europe and Middle Eastern regions,^36^ which are characterized by overall androgenic traits.^37^ They are roughly defined by relatively high androgen levels, hirsutism, androgenic alopecia, and have a reproductive phenotype with chronic anovulation and subfertility.^38^ These clinical and biochemical characteristics could also be because of non‐classical steroid 21‐hydroxylase deficiency, which is a disorder associated with adrenal hyperplasia and characterized by androgen excess, which is highly frequent (3%‒4%) in people of Ashkenazi origin.^39^, ^40^ In summary, the repeated cases of in/subfertility among women within the Terach descendants described in Genesis could reflect PCOS phenotypes still found in Israeli and Middle Eastern people and be related to a polygenic condition and/or partial 21‐hydroxylase deficiency, conditions known to be still highly prevalent among Jews.

PCOS affects exclusively women, although the existence of a PCOS male phenotype has been hypothesized, consisting in subjects carrying PCOS‐related gene variants and hyperandrogenic traits.^41^ From an evolutionary point of view, hyperandrogenism among humans could be a case of *intralocus* sexual conflict, by which the two sexes have different fitness from the same trait.^42^ For instance, while hyperandrogenism leads to PCOS and subfertility in women, it could be advantageous in men, where the trait could be associated with greater strength and aggressiveness. These could have resulted in an evolutionary benefit for males, at the expenses of women’ reproductive fitness.^36^, ^43^

On the male side, both Abraham and, especially, Jacob were very prolific, while Isaac was apparently monogamous and had only two sons. Genesis does not talk about attempts of Isaac of fathering children with other women but informs us that he became father late (subfertility also on the male side? He was the son of consanguineous parents) and then blind. The variable combination of blindness and infertility could be related to vitamin A deficiency,^44^ retinoid signaling defects,^45^ chlamydia infection,^46^ ciliopathies^47^ or the syndrome known as “coloboma, heart defect, *atresia choanae*, retarded growth and development, genital hypoplasia, ear anomalies/deafness” (CHARGE).^48^ A remote possibility could be that he was affected by the Richner‒Hanhart syndrome, a rare autosomal recessive disease, described in a consanguineous Ashkenazi family.^49^ People affected by the syndrome have high levels of free tyrosine and mainly suffer from photophobia, hyperkeratosis of the palms, and soles and slight neurological abnormalities,^50^ linking some traits of the disease with Isaac's blindness. This syndrome, however, is not associated with subfertility. While in the case of Isaac, an infectious disease is of course possible, we have no elements to conclude in favor of any of the above‐mentioned hypotheses and can remain with the posit that he was simply monogamous, his blindness was age‐related, for example, because of cataract, and infertility was confined to Rebekah.

From a secular point of view, the history of Abraham and his family should be regarded as a fairy tale, while from the religious side, the emphasis on the very old age of Abraham and Sarah when they conceived Isaac is to emphasize that everything is possible with God. From a medical point of view, we know now, on the one side, that advanced maternal age is related to aneuploidies and increase of miscarriages.^51^ On the other side advanced paternal age could lead to sex chromosome aneuploidies,^52^ and to epigenetic changes in the spermatozoa resulting in, for example, increased risk of neurological disorders in the offspring.^53^ These were obviously not remarkable problems for Abraham and his wife, at least from what we can read in Genesis. However, the text was written only after many centuries of oral tradition and, admittedly, some “reporting bias,”^54^ omitting negative effects of old age, could have taken place.

## 6 EPILOGUE

Couple infertility is a medical problem as old as the hills and humans were always frustrated by this condition. The bible is one of the oldest written testimonies of this frustration and of the measures taken to address the problem in times when medical assisted reproduction was not possible. These included prayers, herbal remedies, and surrogate womb pregnancies, tools still incredibly popular and currently in use.^55^, ^56^, ^57^, ^58^ In the bible, infertility is always a female problem and in many areas of the globe this belief is still en vogue and male infertility is viewed as an emasculating condition shrouded in myth and requiring divination.^59^ Not much progress from the times of Genesis indeed. Of note, in the bible, when bareness was overcome through divine intervention, it was always to raise up a great prophet and this can be secularly regarded as the great joy that follows the birth of a son/daughter much sought after, which is the common experience of couples successfully attending infertility treatment.

Remarkably, the bible reports several examples of infertility, but it is only in the case of Abraham's family that Genesis gives enough details to attempt a secular, medical interpretation of the problem. Analyzing this tale, we offer a medical “exegesis”: the Terach's descendants were affected by a polygenic form of PCOS or by partial 21‐hydroxylase deficiency. To remain at the border between the serious and the facetious, we could even speculate that Abraham's family was the “founder” of the Mediterranean phenotype of this condition. This is of course something that cannot be proven and should remain in the creative and imaginative spirit that was adopted to compile this paper. The authors hope that their interdisciplinary approach may be appreciated by the few curious readers whose attention it will attract.

## AUTHOR CONTRIBUTIONS

Manuela Simoni performed the article research, conceptualization, and manuscript writing and editing. Frank Tüttelmann contributed to conceptualization, interpretation of the data, and revised critically the paper. Livio Casarini contributed to conceptualization, research, data interpretation, and manuscript writing and editing.

## ACKNOWLEGMENTS

This paper is dedicated to Ebo Nieschlag, the great promoter of andrology and mentor of dozens of andrologists spread around the globe. He should be regarded as the “patriarch” of “descendants as numerous as the stars in the sky.” His spirit will be always with us. His soul will appreciate this script.

## CONFLICT OF INTEREST STATEMENT

The authors declare no conflicts of interest.

## DISCLOSURE

The authors declare that they have not used AI technology to produce contents of this manuscript.

## Data Availability

Data sharing is not applicable to this article as no new data were created or analyzed in this study.
